# Payment mechanisms to improve prevention spending in health care settings: A policy-focused evidence brief

**DOI:** 10.1016/j.puhip.2026.100756

**Published:** 2026-02-19

**Authors:** Ian Holdroyd, Adnaan Ghanchi, Amy Dehn Lunn, Patrick Diamond, Sashika Harasgama, Helena Painter, Helen Pearce, Payam Torabi, Alice Vodden, Yan-Ling Wong, John Ford

**Affiliations:** a Wolfson Institute for Population Health, Queen Mary University of London, UK; b Department of Public Health and Primary Care, University of Cambridge, UK; c School of Politics and International Relations, Queen Mary University of London, UK; d The Aberfeldy Practice, UK; e University College London Hospitals, NHS Foundation Trust, UK; f Institute of Health Sciences Education, Queen Mary University of London, UK

**Keywords:** Preventative healthcare, Health payment mechanisms, Capitation funding

## Abstract

**The policy challenge:**

Decades of evidence indicate that preventative healthcare is both effective and value for money in improving population health long-term. However, healthcare organisations often focus on short-term factors. Leveraging funding mechanisms to promote prevention may facilitate higher implementation of prevention initiatives. The most common funding mechanisms include capitation, Fee-for-service (FFS) and Pay-for-performance (P4P). Here, we explore the policy relevant studies which explore payment incentives for improving long-term population health and under which circumstances these incentives should be encouraged.

**Key evidence to inform policy:**

There are six key lessons for policy makers from the evidence base of how healthcare funding mechanisms can be best designed to promote long-term prevention: 1) spending on prevention is highly efficient and its beneficial effects are likely underestimated, 2) well-funded capitation models best incentivise preventative healthcare, 3) variable funding mechanisms, such as FFS and P4P, can be beneficial in increasing quantity focused preventative interventions-those with a uniform and straightforward approach, such as immunisation programs, 4) non-payment to hospitals may be effective in encouraging prevention, 5) Social Impact Bonds may facilitate prevention spending whilst mitigating financial risk away from the public sector, but come with complexity, 6) return on-investment tools can improve spending efficiency, but their impact on decision making is unclear.

**Further considerations and implications:**

To most effectively incentivise preventative healthcare, capitation payments should be complemented by more variable funding models for specific preventative services. Alternative payment mechanisms, such as Social Impact Bonds and non-payment may have their place. Integrating equity into decision making shows promise in further improving outcomes.

## Highlights

• Investing in prevention improves population health and is cost effective, but funding structures often prioritise short-term outcomes instead.
• Capitation models are most effective for incentivizing long-term, complex prevention interventions, while fee-for-service (FFS) and pay-for-performance (P4P) models can enhance quantity-based prevention efforts, such as immunisations.
• Social Impact Bonds (SIBs) can enhance prevention spending, though they introduce complexity and risks.
• Return-on-investment (ROI) tools can improve spending efficiency, but their impact on decision-making remains unclear.

## 1 Current policy challenges

Overwhelming evidence shows that investment in prevention improves population health outcomes significantly [[1], [2], [3], [4], [5], [6], [7]]. Public health spending is a notable example. A study in California found that each additional $10 per capita spent on public health reduced all-cause mortality by 9.1 deaths per 100,000 people [1]. Data from England indicated that public health expenditure cost £3,800 per additional quality-adjusted life year (QALY), making it 3.6 times more efficient than healthcare spending (£13,500 per QALY) [2]. Between 2013 and 2019, a 1% increase in English public health expenditure was associated with a 0.15% reduction in multimorbidity prevalence [8]. Furthermore, 85% of the public health recommendations made by the National Institute for Health and Care Excellence were found to be cost-effective [3]. Contrary to popular belief, benefits of spending on prevention can be realised on relatively short to medium term time scales: previous research suggested that most benefits could largely be observed after five years and almost fully realised after eight [1].

Despite the benefits of prevention, the funding structure of healthcare organisations is poorly set up to invest and incentivise preventative health; organisations tend to focus on short-term organisational drivers rather than longer-term preventative programmes. Furthermore, economic analyses often underestimate the cost-effectiveness of preventive spending. A key example is the use of discount rates [9]. Discount rates adjust the present value of expected costs and benefits by considering the time at which they are realised. In the UK, NICE applies a 3.5% discount rate, meaning future benefits decrease in value by 3.5% annually. Discounting reflects the public's preference for immediate health benefits and accounts for the cost of borrowing [10]. However, higher discount rates disproportionately devalue the benefits of long-term interventions, reducing the perceived cost-effectiveness of prevention. Lowering these rates would better support long-term preventative measures.

Prevention is a broad concept with multiple definitions. Here, it is used to refer to any healthcare-based initiative that seeks to improve long-term patient outcomes. This includes healthcare action on lifestyle factors, as well as clinical interventions targeting modifiable risk factors.

Changes to funding mechanisms may be the most effective way to encourage healthcare organisations to implement preventative programmes. Several funding mechanisms are used, usually in combination. The three most common reimbursement mechanisms are capitation, fee-for-service (FFS) and pay-for-performance (P4P) (Table 1) [11]. Here, we summarise evidence on the factors influencing the selection of funding mechanisms to optimise the efficiency of preventive spending.

**Table 1:** Payment mechanisms.

Payment Mechanism	Description	Example
Capitation	Healthcare organisations are paid a fixed fee per patient, regardless of how the services are used.	The Global Sum Payment for Primary Care patients in the UK- annual payments are calculated by multiplying a set fee by the weighted patient on each General Practice's list.
Fee-for-service (FFS)	Organisations are reimbursed for each unit of care that they deliver	A hospital being reimbursed a set national tariff for each flu vaccination that it delivers.
Pay-for-performance (P4P)	Organisations are paid based on quality of provision of care, as measured by specific indicators	A hospital being reimbursed should it ensure that over 95% of patients who smoke are signposted to relevant tobacco cessation services.

## 2 Approach to collating evidence

We undertook a rapid review of the policy-focused evidence to explore funding mechanisms that promote prevention. We focused on healthcare organisations in high-income countries, as defined by the World Bank, and payment mechanisms or tools which increase prevention. A broad range of policy outcomes were considered: 1. patient access, experience, and health outcomes; 2. health service outcomes, such as hospital admissions and emergency department attendances; 3. cost-benefit outcomes; 4. broader societal outcomes, such as employment and benefit uptake; and 5. measures of inequality across these categories.

Our aim was to identify and concisely synthesise the most robust and relevant evidence to inform policy and practice. To do this we undertook a search strategy with snowballing, then prioritised the most relevant and robust studies. This meant identifying studies where policy relevant data was collected and analysed in a sufficiently robust manner. We searched MEDLINE on August 15th, 2024, and identified 576 studies. Search terms and inclusion and exclusion criteria are available in Supplementary File 1. Additional papers were identified through the Health Equity Evidence Centre Living Evidence Maps [12]. The titles and abstracts of each article were independently screened by two authors. Citations of relevant articles were reviewed, and a grey literature search was undertaken using a combination of search engine indexes and searching relevant websites (e.g. The King's Fund). This process identified 51 policy relevant articles and grey literature reports. The following information was extracted from each study: first author, year of publication, aim, type of study, number of studies included (if the study was a review), data sources, time of analysis, population, intervention, comparator, main findings. Due to the large amount of data heterogeneity and the policy focus of this review, it was inappropriate to perform a meta-analysis or quantitatively compare study's findings. Instead, studies were synthesised narratively. The relative weighting applied to each study's findings was based on relevance and scale. Highest priority was given to systematic reviews, relevance of the evidence within the study to funding mechanism, and those conducted at a large scale. Lower priority was assigned to primary studies, smaller studies, or those with limited relevance to the research question.

## 3 Key evidence to inform policy

There are five key areas of learning for policy makers in relation to funding mechanisms that best promote prevention.

1. Capitation funding supports the delivery of more complex, longer-term, preventative care, provided funding is sufficient.
2. Variable funding mechanisms can be beneficial in increasing shorter-term *quantity* focused preventative interventions.
3. Non-payment to hospitals may be effective in preventing adverse events.
4. Social impact bonds can increase preventive funding, foster innovation and reduce risk.
5. Return on Investment tools identify areas of highest efficiency for spending, but their impact on decision-making is unclear.

### 3.1 Capitation funding supports the delivery of more complex preventative care, provided funding is sufficient

**Fig. 1:**
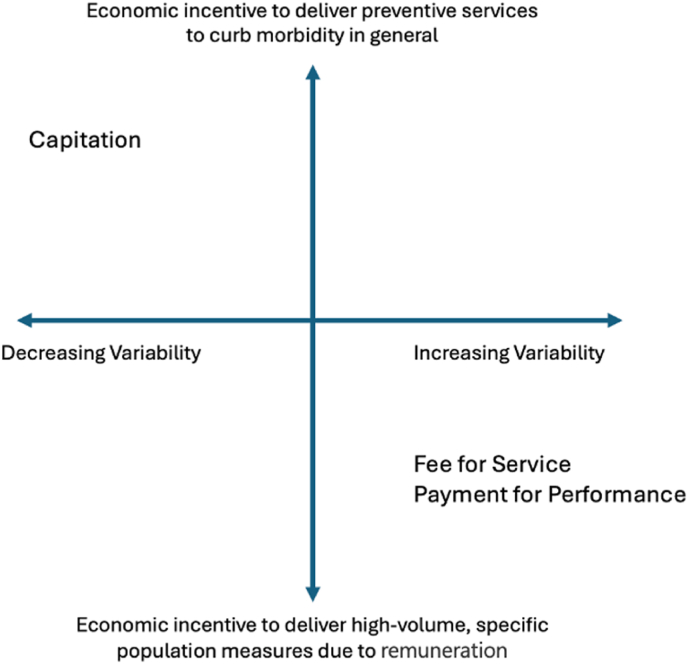
Economic incentives to provide preventative healthcare by payment variability.

The ability of a payment mechanism to economically incentivise long-term preventative care depends on its payment variability, i.e., the degree to which payments fluctuate based on activity. Generally, as payment variability increases, the incentive to provide long-term preventative care decreases [13]. Fee-for-service (FFS) or pay-for-performance (P4P) are systems with a high degree of payment variability. Providers are financially incentivised to deliver preventative care only when specific reimbursements are available for those services. In contrast, capitation has low variability. This incentive structure ensures providers prioritise prevention to reduce their own long-term costs (Fig. 1).

Observational evidence supports this theory. Payment models with lower variability, like capitation, are more effective at incentivising complex and long-term preventive care [6]. A Canadian study found that patients in capitation plans were more likely to receive diabetes monitoring and three types of cancer screening than those in FFS plans. Another study found that over ten years, greater improvements in diabetes and cervical cancer screening were observed in patients under capitation models [14]. A third found that patients treated under a plan blending capitation and FFS had better outcomes for diabetes and obesity prevention and were more likely to receive smoking cessation support compared to those in FFS plans [15]. Finally, a US study found that general practitioners whose reimbursement consisted of more than 75% capitation, as opposed to FFS, were three times more likely to provide patient education [6,16].

We did not find evidence exploring factors that influence the effectiveness of capitation plans in promoting prevention. One notable limitation of capitation is its reliance on sufficient funding. When funding is constrained, priorities often shift toward acute, reactive care rather than long-term prevention.

### 3.2 Variable funding mechanisms can be beneficial in increasing *quantity* focused preventative interventions

Highly variable funding mechanisms include FFS and P4P. Some studies showed that FFS is more effective in increasing preventative healthcare quantity. For example, the most robust evidence for both FFS and P4P pertains to vaccination [6,7]. Two systematic reviews, one including meta-analysis, reported increased vaccination rates following the introduction of P4P programmes [6,7]. These reviews found that FFS models improved immunisation rates, with stronger evidence supporting higher FFS payments.

A Cochrane review found that P4P improved childhood immunisation rates by 27% compared to capitation or salaried models. P4P increased guideline-driven prescribing of antihypertensives by 7%. The success of these schemes depended on striking a balance between the size of the financial incentive and the effort required to meet the targets [7].

Evidence on the impact of FFS and P4P on screening was limited. One systematic review suggested that P4P schemes could improve screening rates, with more significant effects observed when P4P was directed at individual physicians (a 60% to 145% increase in screening rates) compared to practices (a 2.4% to 11.0% increase) [17]. One review reported limited or no effects of financial incentives on breast and cervical cancer screening, but some positive or partial effects for colorectal cancer screening [18]. Of three studies investigating the effects of increased FFS payments on screening, two associated higher reimbursement rates with improved screening, while one found no link [17]. A UK study examining the impact of a large P4P scheme and cervical screening found a decline in screening rates [19].

Much evidence regarding P4P is derived from the Quality and Outcomes Framework (QOF), a UK scheme that reimburses general practices based on performance against 76 indicators, most of which measure preventative care quality. While initial evidence suggested positive impacts [20,21], subsequent analyses have yielded mixed results. In many cases, improvements in preventive care preceded the introduction of the QOF, and post-implementation results did not consistently show sustained progress [[22], [23], [24]]. Furthermore, its impact on long-term health outcomes is limited, with studies indicating that the QOF was not associated with reductions in mortality [25].

Concern exists regarding the sustainability of such schemes. For example, Scotland discontinued the QOF, citing its outdated and overly bureaucratic nature [26]. Following its removal, performance in 75% of previously incentivised indicators worsened [26]. This highlights their lack of long-term effectiveness: performance declines when indicators are removed, suggesting that the QOF did not embed lasting changes in practice amidst competing resource pressures [27].

Other potential drawbacks of FFS and P4P described in the literature include.

• Both models constrain care delivery to predefined standards, reducing the flexibility needed for certain types of preventive care [6].
• Achieving P4P targets does not always translate to improved health outcomes [22,25,28,29].
• FFS and P4P may lead to the prioritisation of incentivised tasks at the expense of other important but non-incentivised preventive measures [29]. For instance, care for conditions outside the QOF has worsened since its introduction [24,30].
• Improvements under FFS and P4P are often time-limited and fail to drive long-term changes [6,7,26].

### 3.3 Non-payment to hospitals may be effective in preventing adverse events in hospitals

Non-payment, rather than financial rewards, can effectively reduce rates of adverse medical outcomes. A Cochrane review examined evidence on this approach, focusing on US schemes that penalised hospitals for failing to meet quality targets related to surgical site infections, catheter-associated infections, antimicrobial use, hospital-acquired pressure ulcers, and inpatient falls [31]. These schemes were associated with reductions in adverse clinical events, with impacts larger than those observed in studies evaluating financial rewards for good performance. Another study investigating surgical quality suggested that pay-for-performance programmes incorporating penalties were more effective than those relying solely on rewards or a combination of rewards and penalties [32].

### 3.4 Social impact bonds can increase preventive funding, foster innovation and reduce risk

Social impact bonds (SIBs) are financing mechanisms in which private investors fund preventative programmes upfront, with repayment by the government or other organisations contingent on achieving predefined outcomes for a designated population. Repayments may be partial or complete depending on the level of target outcomes achieved [33]. In theory, SIBs transfer financial risk from the public to the private or not for profit sector, with payments made only when outcomes are successfully achieved [34].

A global review examined 11 SIBs targeting non-communicable diseases across New Zealand, Canada, the United Kingdom, the USA, Japan, Israel, Australia, and the Netherlands [35]. Overall findings were limited by the small sample size. Of the four completed SIBs, three met or exceeded all targets, while one met some targets. Among ongoing SIBs at the time of review, two were partially meeting targets, one was below target, and four had an undisclosed performance. Key success factors included evidence-based interventions, collaboration with multiple service providers, and the involvement of intermediaries to coordinate between contractors and suppliers.

However, potential drawbacks of SIBs noted in the literature include: 1. The cost of negotiating SIBs may outweigh the financial savings achieved [35]; 2. Instances of “cream-skimming” have been reported in which private investors select interventions or work with target populations with the highest likelihood of success, thereby increasing the likelihood of generating a financial return [35]; 3. Performance-based outcomes may incentivise gamesmanship [36]; and 4. SIBs may lead to the marketisation of the public sector with negative repercussions for disadvantaged groups, altering the relationship between healthcare and citizens [37].

### 3.5 Return on investment tools identify areas of highest efficiency for spending, but their impact on decision-making is unclear

Return on-investment (ROI) tools quantify the resource and financial implications of implementing interventions at both national and local levels, helping identify the most cost-effective options. Examples of publicly available ROI tools include those for musculoskeletal diseases, sexual and reproductive health, and colorectal cancer [[38], [39], [40]]. However, no studies were identified that specifically measure the impact of implementing ROI tools in local decision-making.

## 4 Limitations of the literature

The evidence on prevention spending was primarily linked to immunisation, screening, and medical management. It is difficult to draw lessons in other areas of prevention as it is possible that payment mechanisms may operate differently across different domains. The rapid nature of this policy-focused evidence review raises the possibility that relevant studies may have been missed. It is possible that the rapid review methodology introduced bias into the overall conclusions. In particular, the streamlined search and screening processes and limited database coverage increase the risk of selection and publication bias. While we prioritised evidence from systematic reviews, large studies, and methodologically robust research, a formal risk-of-bias assessment was not undertaken, as this was not compatible with the rapid review methodology. A full systematic review would be expected to reduce these risks. We aimed to mitigate these risks by double-screening articles, searching a wide range of sources, and involving multiple researchers who independently interpreted the findings. The review team included researchers from different academic backgrounds to reduce the risk of interpretive bias and to maximise the likelihood that key articles were identified. Additionally, the inclusion of an online evidence map, extensive citation screening, and grey literature searching broadened the range of studies considered. We focused on building a sufficient evidence base for policymakers rather than attempting to conduct a comprehensive review of all relevant studies in a broad and complex topic. Funding mechanisms are impacted by national policy and contractual changes, making outcomes highly contextual and challenging to measure using comparative study designs, which are traditionally considered the most rigorous.

## 5 Recommendations for policy

The findings have significant implications for policy. To most effectively incentivise preventative healthcare, capitation payments should be complemented by more variable funding models for specific preventative services. However, these should only be employed when the benefits of increasing the quantity of such services outweigh the notable drawbacks detailed above. Novel approaches, such as non-payment schemes or social impact bonds, may also hold promise for enhancing preventative care.

When determining funding priorities, evidence from England highlights the importance of integrating health equity considerations to maximise outcome efficiency of any decision. For example, increased national healthcare funding in 2002, adjusted for clinical need, led to a substantial reduction in overall mortality and inequalities in mortality rates. These improvements were predominantly driven by gains in more deprived areas [41]. Adjusting general practice funding based on deprivation data could further enhance funding efficiency, particularly by targeting non-capitation funding streams [42]. Although outside the scope of this review, it is vital to consider the equity effects of these payment mechanisms, which have been examined elsewhere [43,44].

This study is constrained by the evidence available, and the research base would benefit significantly from the further expansion into five key areas. These include 1. Further evidence on the effects of funding variability on preventative healthcare outcomes; 2. Factors influencing the effectiveness of specific funding models, such as capitation, fee-for-service (FFS), and pay-for-performance (P4P), in promoting prevention; 3. In-depth studies on social impact bonds and return on-investment tools and their applicability in healthcare; 4. Economic analyses comparing payment mechanisms, such as cost per QALY or cost-benefit ratios, to inform funding decisions; and 5. Studies exploring the impact of Return on- Investment tools.

## Ethics statement

Not applicable, as this study is based on a literature review and does not involve human participants.

## Availability of data and materials

The data supporting this article's conclusions are available in the referenced studies.

## Authors' contributions

IH and JF conceptualised the study. IH, AG, ADL, SH, HPe, AV and HPa conducted the literature review and data extraction. PD and PT helped interpret the findings. IH and JF led the manuscript writing. All authors contributed to and approved the final manuscript.

## Disclosure statement

The views expressed in this publication are those of the author(s) and not necessarily those of the NHS England, or the Department of Health and Social Care.

## Funding

The research that informed this study was completed in an evidence brief comissioned by NHS England.

## Declaration of competing interest

The authors declare the following financial interests/personal relationships which may be considered as potential competing interests:This report is independent research supported by the National Institute for Health and Care Research ARC North Thames and NHS England. The views expressed in this publication are those of the author(s) and not necessarily those of the National Institute for Health and Care Research, NHS England, or the Department of Health and Social Care.
